# Abortion Care Services and Restrictions in Teaching Hospitals

**DOI:** 10.1097/og9.0000000000000034

**Published:** 2024-09-19

**Authors:** Jody E. Steinauer, Emily Claymore, Kristin Simonson, Heather Steele, Jema K. Turk

**Affiliations:** Ryan Residency Training Program, Department of Obstetrics, Gynecology, & Reproductive Sciences, University of California, San Francisco, San Francisco, California.

## Abstract

The overturning of *Roe v. Wade* has resulted in substantial abortion access restrictions and clinic closures, highlighting the crucial role of hospital preparedness in delivering abortion and pregnancy loss care. This study explores the capacities of teaching hospitals in providing such care. Although most programs can manage health- and life-threatening situations, there are significant regional differences in service limitations and colleague support. For example, compared with faculty members in programs in protective states, those in restrictive states are more likely to report restrictions on first-trimester care (69.4% vs 10.9%, *P*<.001) and second-trimester care (75.0% vs 12.7%, *P*<.001). These findings underscore the vital role of teaching hospitals in ensuring abortion care access, emphasizing the need for enhanced integration of abortion services and training in hospital settings.

## Abstract 2

Faculty members face varying restrictions on abortion training, affecting patient care, especially as clinics close and hospitals become critical sources for abortion care.

The Kenneth J. Ryan Residency Training Program in Abortion and Family Planning (Ryan Program) was established to facilitate the integration of abortion training into obstetrics and gynecology residency programs. As of October 2022, when this study was done, it had supported 114 U.S. programs, each with routine training but with varying capacities to offer abortion services within their hospitals—many referred patients to and trained residents in freestanding clinics. Considering the effects of the 2022 U.S. Supreme Court *Dobbs v. Jackson Women’s Health Organization* decision on freestanding clinic closures,^1^ the importance of hospital preparedness to provide abortion and pregnancy loss care has increased. This study aimed to explore the status of and barriers to abortion care and training in teaching hospitals in late 2022, soon after the *Dobbs* decision.

## METHODS

Faculty Ryan Program directors at 102 eligible programs (holding Ryan designation for 2 years or more) completed an annual survey in October 2022, which included close-ended questions about abortion care and pregnancy loss services in their hospitals, as well as their perceptions of restrictions and colleague support. We categorized programs as “restrictive” or “protective” based on the Guttmacher Institute^2^ estimates in October 2022 and analyzed quantitative data stratified by region and restriction levels using STATA 18. The University of California, San Francisco, IRB approved the study.

## RESULTS

Ninety-one eligible directors (89.2%) responded, representing 19 programs in the West, 15 in the Midwest, 17 in the South, and 40 in the Northeast. Of these, 36 programs were in restrictive states and 55 were in protective states (Table 1); service delivery and perceptions of restrictions and support varied by region and restrictive status (Table 2).

**Table 1.** Description of 91 Ryan Training Programs in the United States, 2022

Program Characteristic	Ryan Programs
U.S. region of program	
Northeast	40 (43.0)
West	19 (20.4)
Midwest	15 (16.1)
South	17 (18.3)
Located in “restrictive” states*	36 (39.6)
Located in “protective” states†	55 (60.4)

Data are n (%).

* States with “restrictive,” “very restrictive,” and “most restrictive” state abortion policies, according to the Guttmacher Institute, October 2022 (Alabama, Arkansas, Arizona, Florida, Georgia, Idaho, Indiana, Iowa, Kansas, Kentucky, Louisiana, Mississippi, Missouri, Nebraska, North Carolina, North Dakota, Ohio, Oklahoma, Pennsylvania, South Carolina, South Dakota, Tennessee, Texas, Utah, Wisconsin, West Virginia).

† States with “some restrictions/protections,” “protective,” “very protective,” and “most protective” state abortion policies, according to the Guttmacher Institute, October 2022 (Alaska; California; Colorado; Connecticut; Delaware; Hawaii; Illinois; Maine; Maryland; Massachusetts; Michigan; Minnesota; Montana; New Hampshire; New Jersey; New Mexico; Nevada; New York; Oregon; Rhode Island; Vermont; Virginia; Washington; Washington, DC; Wyoming).

**Table 2.** Ryan Program Directors' Perceptions of Hospital-Based Abortion Services, Restrictions, and Colleague Support Overall, by U.S. Geographic Region, and by Guttmacher's October 1, 2022, State Abortion Policy Categorization (N=91)

Perception of Abortion Care at Teaching Hospital	Overall	Midwest	South	West	Northeast	*P*	Restrictive States (n=36)*	Protective States (n=55)†	*P*
Services									
Mifepristone on inpatient formulary	76 (84.4)	14 (93.3)	10 (62.5)	17 (89.5)	35 (87.5)	.06	28 (80.0)	48 (87.3)	.35
Mifepristone on outpatient formulary	78 (86.7)	13 (86.7)	12 (75.0)	15 (79.0)	38 (95.0)	.15	29 (82.9)	49 (89.1)	.40
Early pregnancy loss clinic	68 (75.6)	11 (73.3)	11 (68.8)	15 (79.0)	31 (77.5)	.89	28 (80.0)	40 (72.7)	.43
MUA, outpatient	74 (82.2)	14 (93.3)	13 (81.3)	14 (73.7)	33 (82.5)	.53	32 (91.4)	42 (76.4)	.07
MUA, ED	59 (65.6)	10 (66.7)	8 (50.0)	14 (73.7)	27 (67.5)	.50	25 (71.4)	34 (61.8)	.35
Moderate sedation for aspiration	23 (25.6)	3 (20.0)	4 (25.0)	8 (42.1)	8 (20.0)	.30	6 (17.1)	17 (30.9)	.14
Dedicated abortion care service‡	53 (74.7)	3 (42.9)	4 (57.1)	13 (76.5)	33 (82.5)	.10	8 (50.0)	45 (81.8)	.01
Dedicated OR block time for abortion care	53 (58.9)	9 (60.0)	6 (37.5)	10 (52.6)	28 (70.0)	.15	14 (40.0)	39 (70.9)	.01
Restrictions									
1st-trimester abortion care	31 (34.1)	11 (73.3)	11 (64.7)	4 (21.1)	5 (12.5)	<.001	25 (69.4)	6 (10.9)	<.001
2nd-trimester abortion care	34 (37.4)	11 (73.3)	14 (82.4)	4 (21.1)	5 (12.5)	<.001	27 (75.0)	7 (12.7)	<.001
Staff “extremely supportive” of abortion care§									
OR nurses	28 (30.8)	3 (20.0)	4 (23.5)	7 (36.8)	14 (35.0)	.59	9 (25.0)	19 (34.6)	.31
L&D nurses	8 (8.8)	3 (20.0)	0 (0.0)	2 (10.5)	3 (7.5)	.25	1 (2.8)	7 (12.7)	.10
Clinic nurses	56 (61.5)	7 (46.7)	8 (47.1)	14 (73.7)	27 (67.5)	.20	20 (55.6)	36 (65.5)	.29
Anesthesia staff	26 (28.6)	4 (26.7)	6 (35.3)	6 (31.6)	10 (25.0)	.86	10 (27.8)	16 (29.1)	.85
Department chairs	71 (78.0)	12 (80.0)	10 (58.8)	18 (94.7)	31 (77.5)	.08	24 (66.7)	47 (85.5)	.03
Residency program directors	77 (84.6)	14 (93.3)	12 (70.6)	18 (94.7)	33 (82.5)	.17	29 (80.6)	48 (87.3)	.39

MUA, manual uterine aspiration; ED, emergency department; OR, operating room; L&D, labor and delivery.

Data are n (%) unless otherwise specified.

* States with “restrictive,” “very restrictive,” and “most restrictive” state abortion policies, according to the Guttmacher Institute, October 2022.

† States with “some restrictions/protections,” “protective,” “very protective,” and “most protective” state abortion policies, according to the Guttmacher Institute, October 2022.

‡ Due to survey logic, not all program directors were asked this question. For all other comparisons, at least 90 program directors answered the question.

§ Survey question asked, “How supportive do you consider [position name] of the abortion and family planning rotation?” Respondents were then asked to select one of the following: 1) extremely unsupportive; 2) somewhat unsupportive; 3) neutral; 4) somewhat supportive; 5) extremely supportive.

Directors' reports of in-hospital services for caring for people needing abortion or experiencing pregnancy loss varied: 86.7% reported having mifepristone on outpatient formularies and 84.4% on inpatient formularies, 82.2% could offer patients manual uterine aspiration in outpatient clinics and 65.6% in emergency departments, and 25.6% could provide moderate sedation for in-clinic aspiration. Most of these services did not vary by region or state restriction status. However, directors in protective states were more likely to report having a dedicated abortion care service and having dedicated operating room block time for abortion cases than were those in states with more restrictions.

Overall, 34.1% of directors reported limitations on the first-trimester abortion care they could provide and 37.4% reported limitations on the second-trimester abortion care they could provide, varying significantly by region and restrictive status (Table 2, *P*<.001). Directors indicated that limitations were often due to hospital policies related to legal restrictions on public or state university hospitals. Most program directors indicated they were able to provide some abortion care for medical reasons, including pregnancy loss, pregnancy or health complications, or fetal anomaly, although allowable indications varied.

A majority of respondents reported that their chairs (78.0%) and residency program directors (84.6%) were extremely supportive of abortion care services and training. Perceptions of support by nurses varied by service; most reported that clinic nurses (61.5%) were extremely supportive, but a minority reported the same support for operating room nurses (30.8%) and labor and delivery nurses (8.8%), and only 28.6% reported that anesthesia staff were extremely supportive. Overall, these reports did not vary by restrictive status, with the exception that 66.7% of directors in restrictive states found their chairs to be extremely supportive, compared with 85.5% in protective states (*P*=.03).

## DISCUSSION

Faculty members who oversee abortion training report limitations on services they can provide patients at their training hospitals, with differences in services, restrictions, and colleague support by state restrictive status. As clinics close due to abortion bans, hospitals must be ready to meet the needs of patients who require hospital-based abortion care, for example, those with significant pregnancy complications and for whom it would be especially harmful to travel for care. Furthermore, the closure of clinics should compel teaching hospitals to assume responsibility for providing Accreditation Council for Graduate Medical Education–mandated abortion training^3^ for future obstetrician–gynecologists and to model comprehensive reproductive health care.
